# Psycho-social and environmental correlates of location-specific physical activity among 9- and 15- year-old Norwegian boys and girls: the European Youth Heart Study

**DOI:** 10.1186/1479-5868-3-32

**Published:** 2006-09-25

**Authors:** Yngvar Ommundsen, Lena Klasson-Heggebø, Sigmund A Anderssen

**Affiliations:** 1 Department of coaching and psychology, Norwegian School of Sport Sciences, P.O. Box 4014, Ullevaal Stadion, 0806, Oslo, Norway; 2 Department of Sports Medicine, Norwegian School of Sport Sciences, P.O. Box 4014, Ullevaal Stadion, 0806, Oslo, Norway

## Abstract

**Objective:**

Little is known about the existence of independent location- or context specific forms of physical activity. This study sought to identify location-specific forms of physical activity in a sample of 9 and 15 years-olds Norwegian boys and girls, and examined their associations to psycho-social and environmental factors.

**Methods:**

A cross-sectional study of 9 and 15-year-olds (N = 760; 379 boys and 381 girls) was conducted in which participants responded to a computer-based questionnaire (PEACH) tapping potentially location specific forms of physical activity as well as psycho-social and environmental correlates.

**Results:**

Exploratory factor analysis indicated that the nine and fifteen year-olds self-reported their physical activity as located in three separate and specific contexts: a) school commuting, b) informal games play at school and c) organized sport, structured exercise and games play in leisure time. Dependent of location, psycho-social and environmental correlates explained between 15 and 55 percent of the variance in physical activity. The impact of peer support, enjoyment and perceived competence in physical activity generalized across the three locations. Enjoyment of physical education classes, parental support and teacher support, in contrast, confined to particular location-specific forms of physical activity. Generally, behavioural beliefs and environmental factors represented marginal correlates of all location-specific forms of activity.

**Conclusion:**

Young peoples' physical activity was identified as taking place in multiply genuine locations, and the psychosocial correlates of their physical activity seem to some extent to be location specific. Results may inform intervention efforts suggesting that targeting specific sets of psycho-social factors may prove efficient across physical activity locations, gender and age groups. Others, in contrast may prove effective in facilitating location specific physical activity, in which age may come to moderate the efficiency of intervention efforts.

## Background

Previous research has shown that influences on young peoples' physical activity are multi-factorial. A variety of psychological, social and physical environmental correlates of physical activity for young people have been identified, and social-cognitive models that emphasize intrapersonal, micro-environmental influences and physical activity behaviour, hold great promise for addressing the physical activity participation problem. Young peoples' capability beliefs, affect outcome expectations or behavioural beliefs, parental, peer, teacher/school as well as the social and physical environmental influences impact on young peoples' physical activity [1,2]. For example, it is widely accepted that perceived competence and enjoyment influence young peoples' physical activity, and social support from family and peers have also been identified as positive correlates [3]. Behavioural beliefs such as outcome expectancies of physical activity have, however, been less well researched and existing evidence on their influence are equivocal [4]. Further, we know far less about the extent to which age and gender interact with psychological, social and physical environmental factors in their influence on physical activity.

Despite renewed focus from a public health perspective on school physical education, the possible influence of school specific psychological factors on children and youth's physical activity has received little research attention [5-8]. There is preliminary recent evidence that a teacher supportive climate in PE as well as a strong physical education culture at school influence young peoples' intuitive interest in school physical activity and physical education [9-11]. However, the role of school teachers in stimulating young peoples' leisure-time physical activity is less clear, and more research on the role of teacher social support and enjoyment in PE on young peoples' self-reported physical activity is warranted [9,10].

Research has shown that physical environmental factors such as facility access (e.g. location of parks and physical opportunities to be active) are associated with children's physical activity [3]. In contrast, social environmental influences on young peoples' physical activity such as perceived opportunities in the form of environmental safety and social environmental influences including perceived access to playmates in the neighbourhood and parental restrictions, acceptance and monitoring regarding on being outdoors (licence) have been less well researched [12]. Further examination of a greater variety of perceived physical and social environmental factors has been called for [13].

### Location specific physical activity

Young peoples' physical activity may take place in different contexts or locations in which they operate on a daily basis [3]. For example, they may commute to and from school by foot or by bicycle; they may use school recess for playing games and doing sports; and they may participate in organized competitive sport or otherwise be involved in exercise, physical activity and games in their after-school leisure time. Indeed, physically active commuting to and from school would be regarded as a different form of movement activity than would for example school recess games play or competitive sport after school. Hence, the relative importance of correlates of young peoples' physical activity in these contexts and locations may vary dependent on characteristics of the activities taking place as well as the subjective meaning young people ascribe to those activities [14]. For example, in the case of leisure-time organized sport and performance oriented exercise and games, perceived competence may come to play a stronger role for participation and persistence than would be the case for play-like physical activity in the form of school recess games. In particular, this would be so among the older boys and girls [15]. Moreover, peer support and support from teachers at school would be expected to yield a stronger influence on young peoples' informal games play during school time than would parental support. In contrast, social support from parents and peers would both be expected to be equally important for participation in structured exercise and physical activity in the format of competitive organized sport [3]. The role of school teacher support in stimulating young peoples' leisure-time physical activity is less clear [9,10]. For example, physical education teacher has been shown to represent a marginal significant others in terms of pupils' leisure-time physical activity [16]. Hence, we would not necessarily expect physical activity support from teachers to generalize to leisure-time sport and physical activity. In terms of school commuting, we would expect social peer support to play a role. However, given that structural barriers such as physical barriers hindering walking or rules or regulations preventing cycling may hinder some young people from active commuting, the role of peer support should appear modest. We would expect social environmental factors such as access to play-mates in the neighbourhood to correlate with physical activity across context/location activity formats, whereas the potential importance of physical environmental factors in this respect seems less clear [17].

Hence, by typically treating young peoples' physical activity as a one-dimensional construct using a composite measure of physical activity, potential location specific variations in the impact of influences on young peoples' physical activity may have gone unnoticed in previous research [18,19]. Accordingly, more research is needed to systematically examine the relative importance of different psychosocial and environmental correlates of young peoples' location specific physical activity. Besides, an identification of the extent to which age and gender moderate the role of different location-specific psycho-social and environmental correlates of physical activity has also been called for [3,20]. Clearly, knowledge of the potential interplay of location specific correlates of young peoples' physical activity and age and gender in predicting such activity would be valuable to planners in tailoring interventional efforts.

The main aim of this study, therefore, was to extend previous research by

1) Exploring the existence of different location-specific forms of physical activity.

2) Examining the relative impact of a greater variety of psycho-social and environmental correlates on young peoples' physical activity within and across location-specific forms of physical activity.

3) Exploring whether age and gender moderated the role of physical activity correlates within different location specific forms of physical activity.

In order to conduct age-related comparisons, the use of identical measurement methods for physical activity for all age groups seems necessary. Unfortunately, previous studies have used different measurement tools for different age groups. Studies of older youth have typically used paper and pencil self-reports, while studies on younger children have usually used either objective measures or parent self-report [3]. Admitting that existing paper and pencil based self-reporting methods are of questionable value when used with young children [21], new computer based self-reporting instruments that are potentially more motivating, easy to follow and thus appropriate for children at all ages should be tested [21]. It is essential that understandable concepts of physical activity are used and that the children are able to make valid judgments by the research situation [21,22]. Moreover, as opposed to objectively measured environmental influences, self-report data pertaining to perceptions of the physical and social environment are inexpensive, and they can more easily be included in a variety of populations and locations. Hence, the use of self-report measures makes an evaluation of environmental influences on young peoples' physical activity more feasible [6,7]. Accordingly, in the present study, a computer-based self-report instrument PEACH (Personal and Environmental Associations with Children's Health) was used to assist children and adolescents in reporting their physical activity as well as its correlates. Nevertheless, for validation purposes, the computer based self-reports of physical activity was criterion validated by means of an instrument (e.g. CSA activity monitor) objectively measuring physical activity.

## Methods

### Subject selection and study design

This cross-sectional study was conducted as a part of the European Youth Heart Study [23]. All compulsory primary and secondary schools in Oslo were stratified according to school level and the socioeconomic character of their local areas. Pupils (N = 578) from nine primary schools (4^th^grade, 9-year-old) and 890 pupils from ten secondary schools (10^th^grade, 15-year-old) were invited to participate. The regional ethical committee approved the study, and parents and children signed a written consent before participating in the study. Seventy-five percent of the 9-year-old children and 42% of the 15-year-old youth agreed to participate. The dropout rate was 3.5% among the 9-year-olds and 7% among the 15-year-olds. Thus, a total number of 410 9-year-olds (212 boys and 198 girls) and 350 15-year-olds (167 boys and 183 girls) participated in the study. Observations and tests were performed at the respective schools. Data was collected in small groups of 8–10 children throughout the school year. As part of the data collection 'day' each child completed the computerised questionnaire PEACH. Completion of all questions on the computerised questionnaire took on average 20–30 minutes with children left alone to complete their responses but with a researcher nearby if they needed help. The computerised questionnaire was designed to encourage considered answers and children could not proceed to the next page without completing the relevant answer. A standardised research protocol for the total EYHS was followed in collecting, processing and analysing the data [23].

### Measure of location-specific physical activity

The part of the PEACH developed to measure physical activity comprised nine-item that focused on degree of active commuting to and from school, physical play and informal games play during school recess and right after school, as well as participation in organized sport and physical activity & exercise during leisure time. The questionnaire was modelled and modified based on previously validated self-reports instruments [22]. Hence, this self-report instrument was constructed such as to examine physical activity as scored by an total index, but also to allow for exploring whether physical activity in different daily life contexts/locations could be identified [12,22-24]. All nine questions regarding physical activity were posed without having the pupils think about a particular time frame.

### Correlates of physical activity

#### Perceived physical competence

Perceived physical competence [2] was measured as the sum-score of three items taken from the "Children's attraction to physical activity" (CAPA) scale [25]. These items reflect young peoples' estimation of their physical competence in games play, physical activity, and sport as compared with others of their age. An item example is: "I feel that I am better than most other kids my age at games and sports."

#### Physical activity enjoyment

Enjoyment of physical activity was measured as the sum-score of four of the original five items taken from the CAPA scale [25]. An example of one item is: "Playing games and sports is the thing I like to do best."

#### Enjoyment of physical education (PE) classes

In the present study, one of the original five items from the physical activity enjoyment scale [25] was used separately in order to measure enjoyment of PE classes. The item was "I really like going to PE class at school" *Behavioural beliefs*: Functional and social beliefs regarding expected outcomes of taking part in physical activity [1] were combined and measured using a nine-item scale taken from studies by Saunders and co-workers [26]. An item example is: "If I were to exercise most days, it would give me energy."

#### Peer support

The "Perceived support of physical activity from peers" subscale [1] was based on three items taken from studies by Reynolds and co-workers [27]. An item example is: "How often do your friends ask you to play out or play sports with them?"

#### Parental support

The-five item "Perceived support and encouragement of physical activity from parents" subscale [1] is based on items taken from studies by Reynolds and co-workers [27]. An item example includes: "How often does your mum or dad exercise or play sports with you?"

#### Teacher support

In order to measure social influence and support for physical activity from teachers a three-item scale was developed specifically for the EYHS study [23,24]. The three items were: "How often do your teachers talk about exercise in lessons?", "How often do your teachers organize or play games with you, apart from PE?", and "How often do your teachers tell you to exercise or play sports?"

#### Perceived physical-social environmental influences

Environmental influences were measured through three dimensions, opportunity, facility and licence, respectively:

##### Opportunity

Three items taken from Sallis and co-workers [6] were used to measure perceived opportunities pertaining to physical and social environmental opportunities for being physically active. Two item examples include: "There is somewhere at home where I can go out and play". "It is safe for to walk or play alone in my neighbourhood during the day".

##### Facility

Two items measuring perceived access to playgrounds, parks and gyms in their neighbourhood taken from Sallis and co-workers were used [6]. Items include: "There are playgrounds, parks, or gyms close to my home where I can exercise or play sports", "At school there are playgrounds or fields where I can run around"

##### Licence

We used two items measuring acceptance, monitoring and follow-up by parents of their children to stay out taken from Sallis and co-workers [6]. Items include: "I always have to tell my parents where I am when I go out", "If I am going out, I always have to be back by a certain time".

The items comprising the various correlates had either a 4-point response format (e.g. ranging from hardly ever or never to every day), or a 3-point format (e.g. from definitely yes to definitely no). Support for factorial validity and evidence of reliability for all scales have been reported elsewhere (e.g. in another EYHS study based on pooled data from four different countries in which the current sample constituted on of the four sub-samples) [24]. The correlates included in the EYHS study were theoretically informed and used in previous research, but questions and responses were modified based on feedback from focus groups in the development phase of the EYHS protocol [23]. Hence, given this and that measures generally consisted of few items in order to keep decent length and cover all determinants deemed relevant, values of alpha were generally deemed acceptable according to the rule of thumb by Nunnally (1978) [24]. Indeed, reliability estimates for the total sample (see Table 1) were also shown to be in line with those found for similar constructs in previous studies, in which two-week test-retest reliabilities has been shown to range from .48 to .88 [24]. Generally, the reliability estimates were equivalent for the 9 year-olds and the 15 year-olds.

**Table 1:** Zero-order correlation matrix of dependent and independent variables (total sample, N = 760)

Variable		1.	2.	3.	4.	5.	6.	7.	8.	9.	10.	11.	12.	13.	α
1.	PA 1 (school commuting)	-	.28 ^c^	.03	-.04	.14^a^	.13^a^	.02	.12^a^	.01	.11^a^	.05	-.01	-.11^b^	.72*
2.	PA 2 (school informal games)		-	.10^a^	-.01	.28^c^	.13^a^	.07	.41^c^	.18 ^c^	.32^c^	-.02	.14^b^	-.24^c^	.79
3.	PA 3 (organized sport & PA)			-	.37^c^	.39^c^	.12^a^	.29 c	.41 ^c^	.40 ^c^	.05 ^c^	.19^b^	-.06	.12^b^	.72
4.	Perceived competence				-	.27^c^	.23^c^	.27 ^c^	.21 ^c^	22 ^c^	.01	.12^a^	- .03	.11^b^	.41
5.	PA Enjoyment					-	.24 ^c^	.33 ^c^	.34 ^c^	.29 ^c^	.16^a^	.03	.10^b^	-.05	.65
6.	PE enjoyment						-	.06	.13^a^	.14^a^	.06	.07	.01	-.02	#
7.	Behavioural beliefs							-	.26^c^	.27^c^	.07	.13^b^	- 01	.01	.66
8.	Peer support								-	41^c^	.23^c^	.18^c^	.03	-.08^a^	.78
9.	Parental support									-	.27 ^c^	.09^a^	.05	-.07	.66
10.	Teacher support										-	-.03	.18^b^	-.13^b^	.65
11.	Opportunity											-	- .28^c^	.01	.57
12.	Facility												-	-.10^b^	.29*
13.	Licence													-	.54*

^a^p < .05. ^b^p < .01. ^c^<. 001.

note: # one item measure; * two-item measures (e.g. inter-correlations)

## Statistical analysis

The statistical package for the social sciences (SPSS) version 11.0 was used to conduct all analyses. Preliminary analyses included a description of mean values and zero-order correlations between physical activity and the correlates. An exploratory factor analysis was performed in order to examine whether location specific typologies of physical activity could be identified. In order to examine the relationship of psychological, social and environmental correlates to such location specific forms of physical activity, we conducted hierarchical multiple regression analyses using the sets of correlates as independent variables and the three location specific activity dimensions (e.g. PA 1, PA 2 and PA 3 in Table 1) as dependent variables. Following the recommendation of Sallis and co-workers [7] all correlates were included in the first model specification for PA 1, PA 2, and PA 3, respectively. Then only correlates contributing significantly to each physical activity dimension were included in the re-specifications and presented. In all regressions models, demographic influence comprising parents' education level was controlled for in all analyses (data not shown), and entered in a first block as a statistical control, given previous research indicating these variables to influence young peoples' physical activity [3,7]. Parental education is only presented when significant.

In order to examine possible age and gender interaction effects with respect to the impact of the correlates of physical activity, we included we included measures of age and gender in a separate step, pre-computed interaction terms of each correlate and age and gender, respectively in a last step in each regression analyses. Included were only interaction terms based on those main effects shown to be significant in the re-computed regression models. Prior to specifying interaction terms all included variables were centred round their mean to account for multicollinearity that might result from a high correlation between the first-order terms and the interaction terms if the former is not centred [28].

Following the recommendations of Sallis and co-workers [7], the blocks of correlates (e.g. psychological, social and environmental), was ordered using hierarchical multiple regression models to enhance relevance for intervention design. Hence, demographic information, which was not modifiable, was entered first. Psychological variables were entered second because most intervention efforts to increase physical activity are focused on psychological aspects of individual children and adolescents. Social variables (as perceived by the participants) were entered third, because some type of intervention has targeted change in young peoples' social networks. Environmental variables (as perceived by the participants) were entered last in order to estimate whether any change in physical activity could be expected above the changes accounted for by psychological and social influences.

## Results

### Location-specific physical activity

A principal component exploratory factor analysis with varimax rotation of the nine items aimed at tapping physical activity was performed to examine whether these items reflected forms of physical activity that were location specific. The analysis resulted in the extraction of three factors with an Eigenvalue above 1.0, explaining 49% of the variance in the matrix. Only items with a loading of .50 or above were retained, which resulted in all nine items being included. The item communalities were acceptable (range 0.43–0.86) with no evidence of item cross-loadings. As shown in Table 2, on factor 1 physical play and informal games play during (a) school recess, (b) lunch break, and (c) immediate after school loaded. Hence this factor was labelled *School located informal physical games play (PA2) *The forced choice response format for (a) and (b) included three response alternatives: "Sit down (talking, reading)" (1); "Stand & walking around" (2); "Run around playing games" (3). The response format for (c) included four response alternatives: "From hardly ever" (1) to "Every day" (4). (The fourth response alternative "going home for lunch" was deemed irrelevant in the Norwegian school context, and was not included).

**Table 2:** Maximum likelihood exploratory factor analysis of 9- and 15-year-old children and adolescents' responses to physical activity items (varimax rotation)

	PA 2	PA 3	PA 1	*h*^2^
Games play during school lunch break	.82			0.70
Games play during school recess	.78			0.64
Stay behind after school for games play	.60			0.44
				
Sport and exercise in sport clubs		.83		0.69
Stages of physical activity change		.75		0.58
Leisure time physical activity and games play		.65		0.53
				
Physically active commuting to school			.92	0.86
Physically active commuting from school			.92	0.86
				
Eigenvalue	2.27	1.77	1.25	
% variance	28.39	22.18	15.57	

Note: All cross-loadings generally < .10; h^2^: communalities

On factor 2, three items loaded. The first pertained to participation in organized sport, the second comprised the stages of leisure-time exercise change algorithm [29], and the third informal game play during leisure-time. Factor 2 was labelled *Leisure-time located physical activity (PA 3)*. The two sets of items pertaining to participation in organized sports and informal games play during leisure time both had a four response forced choice format ranging from "Hardly ever or never" (1) to "Every day" (4). The stage of change algorithm comprised a five point response format: "I don't exercise and I don't intend to start"(1); "I don't exercise, but I might start"(2); "I exercise sometimes, but not regularly"(3); "I exercise regularly, but have just started to do so" (4), "I exercise regularly and have for over 6 months" (5), respectively.

Factor 3 consists of two items comprising commuting to and from school. We labelled this factor *School commuting physical activity (PA 1)*. Forced choice response alternatives for both items were "By car/motorcycle" (1); "By bus or train" (2); "By bicycle" (3); "By foot" (4).

Evidence of criterion-related validity of these self-report measures was examined through correlations with objective measurements of physical activity, assessed with the MTI (formerly known as the CSA activity monitor) model WAM 716 (Manufacturing Technology Inc, Fort Walton Beach, FL) [30]. Correlations with informal games play PA (PA2), leisure-time PA (PA3) and school commuting PA (PA1) were r = .20, p < .001; r = .29, p < .01; r = .16, p < .01, respectively. The correlation with informal games play (PA2) and leisure-time physical activity (PA3) was based on an overall MTI score including also counts during the weekend, whereas active school commuting (PA1) was correlated with an overall MTI score that not included counts during the weekend. Strong correlations between the MTI and the self-report measurements should not be expected. Whereas the MTI registers all body movements, the self-report measurements tap purposeful body movements limited to and specified according to the three different locations (e.g. PA1, PA2 and PA3). Clearly, the exploratory factor analysis and the pattern of correlations using MTI as a criterion, attest to the preliminary validity of refining the nine-item composite score of physical activity towards its use also for the purpose of measuring location-specific physical activity.

### Hierarchical regression analyses

To examine multivariate relationships between the correlates and the three identified location specific forms of physical activity (PA1, PA2 and PA3), including also possible interaction effects of age and gender, we conducted three hierarchical multiple regression analyses. Standardized regression coefficients, R^2 ^values total, and R^2 ^change values associated with each step are presented for each location-specific form of activity in Tables 3, 4 and 5, respectively.

**Table 3:** Summary of Hierarchical Regressions examining psychological, social and environmental correlates to active school commuting (PA1) (N = 760)

Predictor		beta^a^	beta^b^	beta^c^	beta^d^	beta^e^	R^2 (adjusted)^	R^2 change (adjusted)^
Step 1:	*Psychological factors*						.04***	.04***
	Perceived competence	- .11**	- .10**	-.09*	-.01			
	Enjoyment	.14**	.12**	.11**	.07			
	PE enjoyment	.13**	.12**	.12**	.10**			
Step 2:	*Social factors*						.06***	.02***
	Peer support		.10**	.10*	.03			
	Parental support		-.09*	-.10*	-.07			
	Teacher support		.09*	.08	.01			
Step 3:	*Environmental factors*							
	Licence			-.09**	-.01		.07***	.01*
Step 4:	*Demographics*						. 15***	.08***
	Gender				-.07*			
	Age				-.32***			
Step 5:	*Interactions*						.16*	.01*
	Age x parental support					-.31***		
	Age x teacher support					.34**		

^a^Standardized regression coefficients without *social, environmental factors and demographics *entered into the regression

^b^Standardized regression coefficients with *social factors *entered into the regression

^c^Standardized regression coefficients with *environmental factors *entered into the regression

^d^Standardized regression coefficients with *demographics *entered into the regression

^e^Standardized regression coefficients for interaction terms

*p < .05 **p < .01. ***p < .001.

note: 15 year-olds & boys coded 1, 9 year-olds & girls coded 0.

**Table 4:** Summary of Hierarchical Regressions examining psychological, social and environmental correlates to school located informal games play (PA2) (N = 760)

Predictor		beta^a^	beta^b^	beta^c^	beta^d^	beta^e^	R^2(adjusted)^	R^2 change(adjusted)^
Step 1:	*Psychological factors*						.10***	.10***
	Perceived competence	-.13***	-.15***	-.13***	.02			
	Enjoyment	.30***	.17**	.16***	.10***			
	PE enjoyment	.09*	.08*	.08*	.02			
Step 2:	*Social factors*						.25***	.15***
	Peer support		.32***	.31***	.19***			
	Teacher support		.20***	.18***	.03			
Step 3:	*Environmental factors*						.28***	.03***
	Licence			-.17***	-.01			
Step 4:	*Demographics*						.54***	.26***
	Age				-.60***			
Step 5:	*Interactions*						.55***	.01*
	Age x enjoyment					-.23***		
	Age x peer support					-.15*		

^a^Standardized regression coefficients without *social, environmental factors and demographics *entered into the regression

^b^Standardized regression coefficients with *social factors *entered into the regression

^c^Standardized regression coefficients with *environmental factors *entered into the regression

^d^Standardized regression coefficients with *demographics *entered into the regression

^e^Standardized regression coefficients for interaction terms

*p < .05 **p < .01. ***p < .001.

**Table 5:** Summary of Hierarchical Regressions examining psychological, social and environmental correlates to leisure-time located physical activity (PA3) (N = 760)

Predictor		beta^a^	beta^b^	beta^c^	beta^d^	beta^e^	beta^f^	R^2 (adjusted)^	R^2 change (adjusted)^
Step 1:	Parental education level	.12***	.13**	.13**	.12**	.13**		.01**	.01**
Step 2:	*Psychological factors*							.26***	.25***
	Perceived competence		.26***	.21***	.19***	.15***			
	Enjoyment		.29***	.21***	.22***	.25***			
	Behavioural beliefs		.12**	.05	.04	.02			
Step 3:	*Social factors*							.36***	.11***
	Peer support			.23***	.22***	.28***			
	Parental support			.20***	.21***	.19***			
	Teacher support			-.10**	-.08*	-.02			
Step 4:	*Environmental factors*							.37**	.01**
	Opportunity				.10**	.07*			
	Licence				.13***	.07*			
Step 5:	*Demographics*							.41***	.04***
	Age					.23***			
Step 6:	*Interactions*							.44***	.02***
	Age x competence						.31***		
	Age x teacher support						-.26***		

^a^Standardized regression coefficients without *psychological *and *social factors *entered into the regression

^b^Standardized regression coefficients with *psychological factors *entered into the regression.

^c^Standardized regression coefficients with *social factors *entered into the regression.

^d^Standardized regression coefficients with *environmental factors *entered into the regression.

^e^Standardized regression coefficients with *demographics *entered into the regression

^f^Standardized regression coefficients for interaction terms

*p < .05. **p < .01. ***p < .001.

#### Active school commuting (PA1)

As shown in Table 3, physically active school commuting was associated with low perceived physical competence (beta = -.11, p < .01), high physical activity enjoyment (beta = .14, p < .01), and school PE enjoyment (beta = .13, p < .01). Further, high support from peers (beta = .10, p < .01), and from teachers (beta = .09, p < .05), and absence of parental support (beta = -.09, p < .05) predicted active school commuting. When entering environmental factors in step 3, strong licence (e.g. parental acceptance and monitoring) was shown to relate negatively to active school commuting (beta = -.09, p < .05). Generally, the influence of the psychological factors in step 1 was upheld when entering the various social support variables into the equation in step 2, and environmental influences in step 3. However, several psychological and social correlates were no longer significant when entering the main effect of age in step 4 (beta age = -.32, p < .001). Indeed, active school commuting is more prevalent among the younger than the older ones. As revealed in step 5, age moderated the role of parental support and age moderated the role of PE teacher support. Further inspection of the interactions by means of the differential directions of the beta weights of the two interaction terms indicated that parents more strongly supported active school commuting among their younger daughters or sons than among the older ones, whereas teachers were more supportive of the 15 year olds' active commuting than the 9 year olds'. The psychological, social, environmental sets of correlates, age and the age related interactions accounted for 16% of the variance in active school commuting. Indeed, age alone accounted for 8% of the total variance explained.

#### School located informal games play (PA2)

As shown in Table 4, informal games play during school recess and right after school related negatively to perceived physical competence (beta = -.13, p < .01), positively to physical activity enjoyment (beta = .30, p < .001), and school PE enjoyment (beta = .09, p < .05). Further, support from peers (beta = .32, p < .001), and support from teachers (beta = .20, p < .001) positively predicted informal games play in the context of school. In terms of environmental factors, strong licence negatively influenced school located informal games play (beta = -.17, p < .001). After entering peer, parental and teacher support into the equation in step 2 and licence in step 3, the influence of enjoyment from step 1 was somewhat reduced, but still significant. The negative influence of perceived physical competence and the positive influence of school PE enjoyment were, however, generally upheld. Further, the effect of teacher support and licence were no longer significant when entering the quite strong main effect of age (beta = -.60, p < .001) in step 4. As revealed in step 5, age also moderated the impact of enjoyment and peer support on school located informal games play. Further inspection of the interaction terms by means of the directions of beta weights indicated that physical activity enjoyment and peer support of physical activity associated more strongly with informal games play at school among the nine year olds than among the fifteen year olds. Altogether, the psychological and social correlates, age and age specific interaction influences accounted for 55% of the variance in school located informal games play. Gender was not found to be a genuine predictor of games play.

#### Leisure-time located physical activity (PA3)

As revealed in Table 5, parental education positively influenced leisure time located physical activity (beta = .12, p < .001). That is, children of parents with a higher level of formal education tended to report to be more involved in organized sport, structured exercise and games play in their leisure time. Further, enjoyment of physical activity (beta = .29, p < .001), perceived competence (beta = .26, p < .001) and behavioural beliefs (beta = .12, p < .01) all associated positively with self-reported leisure time located physical activity, together explaining 25% of the variance in physical activity. When adding the set of social correlates to the equation in step 3 and the environmental correlates in step 4, the total explained variance in leisure time located physical activity rose to 37%. With the exception of behavioural beliefs, which were no longer significant when entering the social and environmental correlates into the equation, the effects of the remaining correlates were upheld (see Table 5). These findings indicate that psychological, social and environmental influences act independently and additively as correlates of leisure-time located physical activity. Age moderated the impact of perceived competence and teacher support on leisure time located physical activity. Closer inspection of the interaction terms by means of the direction of the beta weights indicated that perceived competence was more important for leisure time located physical activity among the 15 year olds than among the 9 year olds. In contrast, the negative beta weight for the age x teacher support interaction term indicate that support from school teachers was a stronger correlate to self-report of leisure time located physical activity among the 9 year olds than among the 15 year olds. Altogether, the psychological, social, environmental factors, age and the age specific interaction influences accounted for 44% of the variance in leisure time located physical activity. Gender was not found to be a genuine predictor of leisure time located physical activity.

#### The relative association strength of the correlates across the three physical activity locations

A comparison of the set of psychological correlates versus the social set across the three different locations of activity (see Table 3, 4 and 5), indicates that the amount of variance accounted for by the psychological correlates surpass the amount accounted for by the social correlates both with respect to active school commuting (e.g. 4% versus 2%) and leisure-time located physical activity (e.g. 25% versus 11%). The opposite tendency seems to exist for school located informal games play (e.g. social correlates 15% and psychological correlates 10%). Further, physical and social environmental correlates seem equally marginal across all three physical activity locations, accounting only for 1–3% of additional variance.

When comparing the relative strength of the various psychological and social correlates across the three physical activity locations, similarities as well as differences in relative impact occur. As pairs of predictors, physical activity enjoyment and perceived physical competence generally seem of equally strong relative importance both with respect to active school commuting (enjoyment beta^b ^= .12; perceived physical competence beta^b ^= -.10) as with respect to school located informal games play (enjoyment beta^b ^= .17; perceived physical competence beta^b ^= -.15) and with respect to their relation to leisure-time located physical activity (enjoyment beta^c ^= .21; perceived physical competence beta^c ^= .21). It should also be noted that perceived competence correlated positively with leisure-time located activity, but negatively with informal games play. Enjoyment in physical education classes (beta^b ^= 13), is only marginally stronger related to active school commuting than are perceived physical competence (beta^b ^= -.11), whereas perceived competence (beta^b ^= -.13) seem somewhat more important than PE enjoyment (beta^b ^= .09) with respect to school located informal games play.

Generally, as revealed in Table 3, 4, and 5, beliefs regarding physical activity outcomes seem of marginal importance for physical activity in all three locations of physical activity. Even though results indicate that outcome beliefs act as a genuine predictor of young peoples' leisure-time located physical activity in a first step (beta^a ^= .12, p < 01), the influence of beliefs are attenuated and no longer significant when entering the social correlates (beta^b ^= .05, p >05.), and environmental correlates (beta^c ^= .04, p > .05) into the equation. Table 3 and 5 further reveal that social support from peers and parents (although reversed for parents in the case of school commuting) hold the same relative importance for active school commuting (social support from peers; beta^b^= .10, and social support from parents; beta^b ^= -.09), as for leisure-time physical activity (social support from peers; beta^c ^= .23 and social support from parents; beta^c ^= .20). In contrast, (see Table 4), social support from peers (beta^b ^= .32) and social support from teachers (beta^b ^= .20) are clearly more influential than social support from parents (data not shown) with respect to school located informal games play.

## Discussion

Results from this study provide preliminary evidence that physical activity among young people is best captured by various forms of activity located in three different contexts. Further, in accordance with expectations, several results revealed that young peoples' physical activity seems determined by a complex mixture of psychological and social factors. In contrast, irrespective of location of activity, physical and social environmental factors seem marginally associated with physical activity among these nine and fifteen year olds. Results also reveal that age along side the various correlates accounted for a considerable amount of the variance explained both in school commuting and in informal games play at school. Further, it seems that age for example attenuates the negative associations of licence to school commuting. Apparently, and probably not surprisingly, with increasing age the facilitative effect of low parental monitoring and follow-up on children's active school commuting diminishes as they get older. Not surprisingly as well, physically active school commuting seems less prevalent among the secondary school pupils than among those attending primary school, and informal games play during school seem more prevalent among younger than among the older ones. Possibly more interesting though is the findings that age also seemed to moderate the associations of particular psychological and social correlates to all three location specific forms of physical activity.

The identification of the three different locations for physical activity seems essential given that all these formats of activity are potentially important opportunities to raise young peoples' daily physical activity [11,31-33]. Further, there is evidence that amount of activity in one location may generalize to other locations, and that active school commuting and physical activity during school recess are particularly important in order to establish daily physical activity [31,32].

Generally, *enjoyment *of physical activity, games and sport was shown to be a strong correlate of physical activity involvement both in terms of school commuting, school located informal games play and leisure located forms of physical activity. Age related interaction findings indicated that enjoying physical activity is a stronger correlate of games play among the nine year old boys and girls than among the older ones. Whereas previous research has shown that enjoyment is consistently associated with participation in leisure-time physical activity and commitment to sport [34-36], enjoyment of physical activity apparently also seems to play a role for young peoples' informal games play at school as well as for walking or bicycling to and from school. Second, also *enjoying school physical education *classes associated positively with physically active school commuting and school located informal games play. With respect to school commuting, teacher influence seems important irrespective of age and gender. Hence, physical education teachers may also have a role to play in stimulating physical activity outside PE [10].

*Peer social support *has been shown to be a consistent correlate of physical activity in different samples of young people [37-40]. The present results add to these findings by showing that social support from peers stood out as an important correlate of physical activity across all three different locations, in particular school and leisure-time located activity. One explanation could be that social support from friends for physical activity in one domain reinforces social motivation for being active, which then generalize as a motivational force across location given that physical activity has a strong social asset [31,32]. Nevertheless, social support from peers was less strongly associated with young peoples' commuting to and from school, than with their informal games play at school. Possibly, the degree to which young people are active school commuters may be more controlled by structural hindrances such as busy road barrier or tempting alternatives facilitating inactivity such as good connectivity to school than are informal games play during school-time, for which social factors like peer support may be a more readily influence [17]. The finding showing that peer social support is a positive correlate also of leisure-time located activity attests to the value of efforts to strengthen social group dynamics which may lead to greater persistence in exercise, games play and organized sport in leisure time. Coaches in competitive sport may be particularly valuable in stimulating feelings of social relatedness and reinforcing peer support such as to increase intrinsic and enduring motivation [14,15,41,42].

*Perceived physical competence *was found to be a genuine predictor of physical activity across all three locations of physical activity, in particular with respect to leisure-time located activity. The negative relation of competence to school located games play however, may reflect that the school recess with normally less explicit focus on physical prowess and skills than leisure-time physical activity and sport, are perceived by the less physically competent young ones' as a viable context for being physically active. Findings extend previous ones that primarily has investigated the role of perceived physical competence for participation in activities in which requirements for physical-motor skills are high such as competitive sport [43,44]. The significant positive perceived competence-age interaction for participation in organized sports, structured exercise and games, suggests that perceived competence becomes increasingly more important with age for involvement in leisure-time located context for physical activity. With increasing age then, this may in turn leave less room for those perceiving themselves as less motor-physically skilled [45-47]. It is encouraging though that by creating a mastery-oriented motivational climate in which incremental conceptions of ability and a task orientation can be developed, young peoples' ability perceptions may be upheld [48-50].

Supporting previous research, perceived *parental support *also associated positively with leisure-time located physical activity controlling for other psychological and social correlates [51,52]. Parental support did not however, influence young peoples' school located games play. Apparently, recess play and other forms of school located physical activity are more strongly influenced by context-specific situational reinforcements such as social support from peers. Interestingly, teachers, as opposed to parents, were found to be more encouraging of physically active commuting among the older than the younger group. For safety reasons the nine year-olds, as in the current case, are generally regarded as being too young to be allowed by school authorities to cycle to and from school. Teachers may therefore, more so than parents, set rules and regulations for the younger ones that to some extent may discourage them at least from cycling to and from school. Results pertaining to teacher encouragement of the younger ones with respect to informal games play is important, and reveal that the school may play an important role also in stimulating interest for school located physical activity aside of physical education lessons [3,10], At least it seems that children who report that teachers organize or play games together with them, or talk about the importance of play and exercise in the lesions, are more physically active during school time than their counterparts. Results also reveal that teachers to some extent seem to discourage young peoples' participation in leisure-time sport and physical activity. One could speculate that teachers hold a sport for all perspective, and communicate resistance towards a mainly competitively focused leisure-time sport offers for young people, a setting which often recruits and keep the most talented ones [43,44].

*Functional and social beliefs about outcomes *of physical activity accounted for a significant, although modest amount of variance in leisure-time located physical activity. Further, when controlling also for social and environmental factors, the influence of such beliefs was attenuated. One could speculate that the mixture of social and functional beliefs (including health, and appearance related), embedded in our combined measure may have attenuated a potential influence of any potential sub-dimensions of beliefs. There are findings supporting as well as going against this supposition. On the one hand, recent evidence has revealed that appearance oriented beliefs regarding outcomes of physical activity that reinforce unrealistic and unhealthy norms of physical activity and body image influence 9 to 16 year olds' physical activity [53]. In contrast, studies that has divided beliefs into social and functional ones [24], did not produce any significant differences in influence of social and functional beliefs. The genuine role of different sets of attitudinal beliefs on young peoples' activity clearly deserves more research attention.

Young people's *perceptions of opportunities *in terms of having access to places near by the home to play was positively related to leisure-time located physical activity. Moreover, acceptance and monitoring by parents were found to be negatively related to active school commuting, but positively related to leisure-time physical activity and organized sport. In terms of acceptance and monitoring findings may reflect that whereas parents perceive their children's walking and cycling to school as fundamentally safe, not requiring parental monitoring and follow-up, participation in physical activity and organized sport do require acceptance and monitoring. This may be due to the need of crossing potential unsafe areas and travelling longer distances to reach the places or grounds where their leisure-time located physical activity and sports typically take place. The social-physical environmental factors explained only a marginal amount of explained variance in school commuting and leisure-time physical activity. Previous research that has examined environmental influences on young people's physical activity has revealed equivocal results [e.g., [3,7,13,18,54]]. It should be kept in mind that the influence of the perceived physical and social environment was based on two or three-item measurements, for which estimates of reliability were not particularly strong. Hence, such measures are vulnerable to measurement error which might work to attenuate potential relationships. Irrespective of these methodological reflections on instruments, social-cognitive and ecological models of behaviour suggest that certain aspects of the physical environment can influence participation in physical activity [1,55]. Thus, in future studies, objective measurements of environmental influences should be included together with measures of perceived environmental influences comprising an even greater variety of such aspects that either might facilitate or hinder young peoples' physical activity in different locations. Such work seems underway [56].

## Limitations

Indeed, the limitations pertaining to using even computer-based self-reports of physical activity among the younger ones are admitted. Further, future studies need to confirm the factorial validity of the location-specific sub-dimensions of physical activity by means of confirmatory factor analysis. Due to the nature and difficulty of cross-sectional studies, prospective studies are also needed to provide understanding of causal relationships between physical activity and psychosocial and environmental variables. Further, as already mentioned, the inclusion of measurements with few items may in some instances pose a threat to reliability. Hence, the influence of physical education enjoyment and physical-social environmental correlates may have been underestimated. Also, the results for perceived competence should be interpreted with particular caution in that reliability estimates for this measure were rather low. Clearly, in future research a prospective design should be used in order to better reveal variations in the relative impact of different psycho-social and environmental determinants on contextual/location-specific sub-dimensions of physical activity. Nevertheless, our results attest to recent conceptual frameworks that have been forwarded to understand and examine correlates of physical activity among children and adolescents [57]. Further, as suggested by our findings and recent evidence [58], by supplementing such frameworks to also include measures of location-specific physical activity and measures differentiating between structured and unstructured activity, we may be better able to tease out the most important activity correlates, both within and across locations and contexts of young peoples' activity.

## Conclusion

We identified location specific forms of physical activity among nine and fifteen year old boys and girls. Results reveal that physical activity enjoyment, social support from peers and perceived competence genuinely and quite strongly influenced school and leisure-time located physical activity. Physical activity enjoyment and perceived competence were the correlates most strongly related to leisure-time located activity, whereas peer social support and enjoyment of physical activity related most strongly to school located physical activity, in particular so among the nine year olds. Irrespective of age and gender enjoyment of physical education classes were among the strongest correlates of active school commuting. Teacher support was among the stronger correlates of school located informal games play as was parental social support in terms of leisure-time located activity. Among the younger ones, teacher social support also correlated positively with leisure-time located activity. The psychological and the social sets of correlates both seem equally strongly correlated to active school commuting and leisure-time located activity, whereas the social correlates seems relatively more influential for school located informal games play than the two other location specific forms of activity.

Taken together, the findings highlight the need for physical educators, coaches, parents and public health practitioners to be aware of location specific forms of physical activity, and that both similarities and differences exist with respect to the correlates of physical activity taking place there.
